# The Role of Physical Activity and Exercise in Enhancing Resilience and Delaying Frailty in Saudi Arabia

**DOI:** 10.3390/healthcare13121461

**Published:** 2025-06-18

**Authors:** Abdulaziz M. Alodhialah, Ashwaq A. Almutairi, Mohammed Almutairi

**Affiliations:** 1 Department of Medical Surgical Nursing, College of Nursing, King Saud University, Riyadh 11421, Saudi Arabia; mohalmutairi@ksu.edu.sa; 2 School of Nursing and Midwifery, Monash University, Clayton, VIC 3800, Australia; ashwaq.almutairi@monash.edu

**Keywords:** frailty, physical activity, resilience, aging, older adults, functional capacity, Saudi Arabia

## Abstract

**Background:** Frailty is a major public health concern among older adults, leading to increased morbidity and functional decline. Physical activity and psychological resilience have been identified as potential protective factors against frailty, yet their combined effects remain understudied in Saudi Arabia. **Objective:** This study aimed to examine the associations between physical activity, resilience, and frailty among older adults in Riyadh. **Methods:** A cross-sectional study was conducted with 240 elderly participants aged 60 years and above. Physical activity was assessed using the International Physical Activity Questionnaire (IPAQ), frailty was measured using the Fried Frailty Phenotype, and resilience was evaluated with the Connor–Davidson Resilience Scale (CD-RISC-10). Logistic regression analysis was performed to identify correlates of frailty. **Results:** Higher physical activity levels were significantly associated with greater resilience (*r* = 0.61, *p* = 0.002) and lower frailty (*OR* = 0.64, *p* = 0.002). Advancing age, multiple comorbidities, and poor self-rated health were positively associated with frailty, while greater grip strength was inversely associated with it (*OR* = 0.67, *p* = 0.002). **Conclusions:** Physical activity and resilience appear to be independently associated with reduced odds of frailty among older adults in Riyadh. These findings underscore the potential benefits of integrated physical and psychological health strategies in promoting healthy aging. However, due to the cross-sectional design, causal relationships cannot be inferred. Longitudinal and intervention-based studies are needed to further explore these associations.

## 1. Introduction

Frailty is a multifaceted geriatric syndrome characterized by decreased physiological reserves, reduced ability to cope with stressors, and a heightened risk of adverse health outcomes, including disability, hospitalization, and mortality [1]. As the global population continues to age, identifying effective strategies to delay frailty and preserve functional independence has become a priority in geriatric research and practice [2]. Among the various approaches, physical activity and structured exercise interventions have consistently demonstrated promising results in enhancing resilience, a concept that reflects one’s capacity to adapt, recover, and maintain or regain functioning in the face of stressors such as illness or psychosocial challenges [3]. Understanding how different forms of physical activity foster resilience in older adults can inform evidence-based practices aimed at promoting healthy aging and improving quality of life [4].

Physical activity encompasses a broad spectrum of movements, ranging from leisure-time pursuits such as walking and gardening to more formalized exercise programs that include resistance training or aerobic workouts [5]. Research has shown that older adults who maintain regular physical activity exhibit better cardiovascular function, muscle strength, and balance, all of which are critical components for preserving independence and delaying frailty [6]. Indeed, the physiological underpinnings of frailty—muscle wasting, chronic inflammation, and diminished metabolic reserves—are significantly modifiable through targeted exercise regimens [7,8]. By mitigating these risk factors, regular physical activity contributes to the maintenance of a robust physiological state, thereby enhancing the individual’s resilience against both acute and chronic health stressors [9].

From a mechanistic perspective, structured exercise interventions can attenuate the inflammatory process, bolster neuromuscular function, and improve energy metabolism, all of which are integral to sustaining resilience in aging populations [10]. Resistance training, for instance, has been shown to counteract sarcopenia—a key contributor to frailty—by promoting muscle hypertrophy and enhancing muscle quality [11]. Aerobic exercise, on the other hand, improves cardiorespiratory fitness, increases insulin sensitivity, and helps maintain a healthier body weight, which collectively reduce the risk of cardiovascular diseases and diabetes mellitus [12,13]. The interplay of these physiological improvements not only mitigates the trajectory of frailty but also empowers older adults to better withstand and recover from medical and psychosocial stressors [14].

Beyond its direct physiological effects, physical activity exerts profound psychological and social benefits that collectively bolster resilience [15]. Regular participation in exercise programs has been linked to improved mood, reduced anxiety, and lower rates of depression among older adults, partly due to exercise-induced endorphin release and the psychosocial interaction often inherent in group-based activities [16]. These mental health advantages can translate into a stronger sense of control and autonomy, essential components of resilience that enable older adults to adapt to life changes more effectively. Additionally, group-based or community-centered exercise programs offer avenues for social support, fostering meaningful connections among participants. Such social engagement can buffer the impact of stressors, thereby enhancing individuals’ perceived and actual ability to cope with adversity [17].

One of the critical aspects to consider is the dose–response relationship between physical activity and resilience. Evidence indicates that even moderate-intensity activities, such as brisk walking for 150 min per week, can yield significant health benefits, including reduced risks of chronic diseases, falls, and functional decline [18]. However, identifying the optimal type, duration, and intensity of exercise interventions remains an area of active research. For instance, studies comparing land-based versus water-based exercise programs have reported varying impacts on parameters such as physical performance and body composition, suggesting that intervention choices may need to be tailored based on individual preferences, mobility limitations, and comorbidities [19]. Likewise, the extent to which high-intensity interval training might be safely incorporated for older adults, especially those already exhibiting mild to moderate frailty, warrants further investigation to guide personalized exercise prescriptions [20].

Furthermore, resilience in older adults is not solely determined by physical health. It is a multidimensional construct influenced by cognitive, emotional, and social factors [21]. Physical activity has been shown to support cognitive function by improving cerebral blood flow, neuroplasticity, and the release of neurotrophic factors that protect against age-related cognitive decline [22]. Enhanced cognition, in turn, is integral to resilience, as it empowers individuals to manage health information, adhere to medical advice, and maintain higher levels of autonomy in daily life. In addition, the sense of self-efficacy gained through accomplishing exercise-related goals can promote a more positive outlook, resilience, and an improved perceived quality of life [23].

Despite the recognized benefits, multiple barriers may hinder older adults’ engagement in regular physical activity. These include fear of injury, pain from existing conditions such as osteoarthritis, and limited access to safe, age-friendly exercise environments [24]. Overcoming these barriers typically requires a collaborative, multidisciplinary approach involving healthcare providers, fitness professionals, and community organizations. Tailoring exercise programs to individual abilities and comorbidities, offering clear and accessible guidance, and fostering supportive social networks can significantly enhance adherence and optimize outcomes [25]. Moreover, incorporating behavioral strategies, such as goal-setting and self-monitoring, can further encourage older adults to maintain consistent physical activity habits and thus sustain the resilience benefits over the long term [26].

In recent years, there has also been growing interest in digital and home-based exercise interventions, especially in contexts where mobility or public health restrictions limit access to group programs [27]. Virtual exercise classes, wearable technology for activity tracking, and mobile applications that provide tailored workouts and feedback have shown promise in promoting ongoing engagement and monitoring [28]. However, these digital solutions must address usability challenges among older adults, including comfort with technology and adequate digital literacy. Future research is needed to ascertain whether such innovations can reliably deliver comparable resilience and frailty prevention benefits as traditional, in-person programs [29].

In the context of Saudi Arabia, unique cultural norms and social structures may significantly shape older adults’ experiences of physical activity and resilience. Gender roles, family dynamics, religious practices, and access to age-friendly environments can influence both the willingness and opportunity for older adults to engage in exercise or cope with life stressors. For instance, traditional family support systems may bolster psychological resilience, while gender-specific norms may differentially impact physical activity engagement. Understanding these culturally embedded factors is essential to developing effective, contextually appropriate interventions for promoting healthy aging in Saudi Arabia.

### 1.1. Aim of the Study

This study aims to explore the association between physical activity levels, psychological resilience, and frailty among older adults in Riyadh, Saudi Arabia. Specifically, the study investigates how variations in the type, intensity, and frequency of physical activity are associated with resilience and frailty indicators in this population. By examining these relationships, the study seeks to provide evidence-based insights that can inform the design of culturally tailored, multi-domain interventions to support healthy aging.

### 1.2. Research Questions

1. What are the associations between different types and intensities of physical activity and levels of resilience and frailty among older adults?
2. How are psychological resilience and its subdomains (e.g., adaptability, optimism, emotional regulation) associated with frailty status in community-dwelling and institutionalized older adults?

## 2. Materials and Methods

### 2.1. Study Design

A cross-sectional, observational design was employed to investigate associations between physical activity, psychological resilience and frailty among older adults in Riyadh, Saudi Arabia. Data were collected at a single time point to characterize correlational relationships; causality cannot be inferred from these observations.

### 2.2. Study Setting and Participants

Participants were recruited between February and April 2025 from four types of sites in Riyadh:

1. **Primary Healthcare Centers (PHCs):** Older adults attending routine chronic-disease clinics.
2. **Community and Social Centers:** Independently living, socially active seniors.
3. **Long-Term Care Facilities (LTCFs):** Residents with higher levels of functional dependence.
4. **Rehabilitation/Fitness Centers:** Older adults enrolled in structured exercise programs.

Stratified recruitment across these settings enhanced sample heterogeneity. Informal comparison indicated that LTCF residents exhibited higher frailty scores and lower activity levels, while community-center participants reported greater resilience.

### 2.3. Sample Size and Sampling Method

A target sample of 240 was determined via G*Power (version 3.1.9.7) for logistic regression (medium effect size, OR = 1.5; α = 0.05; power = 0.80), with oversampling to allow for up to 20% attrition. Convenience sampling with voluntary enrolment was used, supplemented by outreach through healthcare staff and community leaders to maximize demographic and functional diversity. Both males and females were recruited in roughly equal proportions.

### 2.4. Inclusion and Exclusion Criteria

- **Inclusion:** Age ≥ 60 years; Riyadh residency; capacity to consent and complete assessments.
- **Exclusion:** MMSE score < 24 (moderate–severe cognitive impairment); terminal illness; unstable medical condition; current enrollment in intensive rehabilitation programs.

Cognitive screening was conducted at enrolment using the Mini-Mental State Examination (MMSE); participants scoring < 24 were excluded to ensure data validity.

### 2.5. Data Collection Instruments

#### 2.5.1. Demographic and Health Questionnaire (Author-Developed)

To capture the contextual profile of participants, the research team created a structured Demographic and Health Questionnaire whose primary aim was to document age, sex, marital status, educational attainment, living arrangement, body-mass index (BMI), number of physician-diagnosed comorbidities, medication use and self-rated health. Items were adapted from widely used geriatric surveys, grouped into two components—sociodemographic characteristics (11 items) and health status (9 items)—and scored as categorical responses, with open fields for height and weight to calculate BMI. Content validity was established by a five-member panel of geriatricians and public-health specialists who judged item relevance (content-validity index = 0.92). Following forward–backward translation, the Arabic version underwent pilot testing in 30 older adults, demonstrating excellent internal consistency (Cronbach’s α = 0.84) and test–retest reliability over a two-week interval (intraclass correlation coefficient, ICC = 0.90).

#### 2.5.2. International Physical Activity Questionnaire—Elderly Version (IPAQ-E)

Originally developed by the WHO-coordinated IPAQ group [30], the IPAQ-E aims to quantify habitual physical activity in adults aged ≥ 60 years. It contains seven items that record frequency (days/week) and duration (minutes/day) of walking, moderate-intensity and vigorous-intensity activities performed during the previous seven days, yielding a composite energy-expenditure score expressed in MET-min/week. Standard scoring classifies respondents as Low (<600 MET-min/week), Moderate (600–3000 MET-min/week) or High (>3000 MET-min/week) activity. Extensive validation studies have shown acceptable criterion validity against accelerometry (ρ ≈ 0.33) and high reliability (pooled test–retest = 0.77). The Arabic IPAQ-E, obtained through forward–backward translation and cognitive debriefing with Saudi elders, retained metric equivalence and demonstrated strong internal coherence (α = 0.87) and two-week stability (ICC = 0.83).

#### 2.5.3. Fried Frailty Phenotype (FFP)

Developed by Fried and colleagues (2001) for the Cardiovascular Health Study [31], the FFP was designed to operationalize physical frailty via five components: unintentional weight loss, self-reported exhaustion, weak grip strength, slow gait speed and low physical activity. Each component is scored dichotomously (present = 1, absent = 0); summing yields a total from 0 to 5, categorized as non-frail (0), pre-frail (1–2) or frail (≥3). Predictive validity for disability and mortality have been repeatedly confirmed (AUC ≈ 0.79), with acceptable internal consistency (α ≈ 0.73). For this study, the tool was translated into Arabic using WHO guidelines; cultural adaptation involved substituting locally understood phrases for “feeling everything is an effort”. A pilot with 40 Saudi elders produced Cronbach’s α = 0.82 and inter-rater ICC = 0.88, supporting reliability of the Arabic version.

#### 2.5.4. Connor–Davidson Resilience Scale, 10-Item Version (CD-RISC-10)

Connor and Davidson (2003) created the CD-RISC to measure psychological resilience; the 10-item short form assesses adaptability, optimism and emotional regulation on a 0–4 Likert scale, yielding total scores of 0–40 (higher = greater resilience) [32]. Meta-analytic evidence shows strong internal consistency (α ≈ 0.88) and construct validity relative to measures of stress and well-being. The Arabic CD-RISC-10 was prepared via dual forward–back translation and expert harmonization, then pilot-tested in 60 community-dwelling Saudi adults (α = 0.89; ICC = 0.86); exploratory factor analysis replicated the single-factor structure, confirming linguistic and conceptual equivalence.

#### 2.5.5. Timed Up and Go (TUG) Test

Devised by Podsiadlo and Richardson, the TUG is a performance-based mobility assessment aimed at predicting falls risk [33]. It entails timing, in seconds, how long a participant takes to rise from a chair, walk 3 m, turn, return and sit; scores ≥ 12 s denote elevated risk. Criterion validity against comprehensive gait analysis is high (r > 0.70) and test–retest reliability excellent (ICC > 0.90). Arabic administration required only translation of brief instructions, verified through back-translation and field rehearsal; reliability in a subsample of 25 Saudi elders was robust (ICC = 0.92).

#### 2.5.6. Handgrip Strength Test

Handgrip dynamometry, standardized by the European Working Group on Sarcopenia in Older People gauges upper-limb muscle strength—a surrogate for overall muscular fitness and frailty. Using a Jamar dynamometer, participants perform three maximal squeezes per hand; the best value is recorded. Reference cut-points for frailty (<30 kg men, <20 kg women) align with international consensus. Criterion validity versus isokinetic testing is strong (r ≈ 0.80), and short-term reliability exceeds 0.95. Arabic instructions, translated and back-translated, were pilot-tested without semantic issues; test–retest ICC in 30 participants was 0.88.

#### 2.5.7. Mini-Mental State Examination (MMSE)

Folstein et al. (1975) developed the MMSE as a brief cognitive-screening tool comprising 30 items across five domains [34]: orientation, registration, attention/calculation, recall and language/visuospatial skills. Scores of 24–30 indicate normal cognition, 18–23 mild impairment and <18 moderate–severe impairment. Extensive validation attests to good sensitivity (0.81) and specificity (0.89) for dementia, with reliability coefficients > 0.85. The Arabic MMSE, produced via licensed forward–backward translation and normative calibration in Gulf populations, retains the original’s psychometric integrity (α = 0.91; ICC = 0.90) and required only substitution of culturally appropriate items for “season” and “serial sevens”.

### 2.6. Data Collection Procedure

The data collection process was conducted systematically to ensure accuracy, consistency, and participant comfort. All assessments were carried out in primary healthcare centers, community senior centers, long-term care facilities (LTCFs), and rehabilitation centers across Riyadh. Trained researchers and healthcare professionals facilitated data collection to ensure that participants understood each assessment and that responses were recorded accurately. Data collection involved multiple steps, including participant recruitment, questionnaire administration, physical activity assessment, frailty evaluation, and functional capacity testing.

The first phase involved participant recruitment and screening. Healthcare professionals, social workers, and community leaders helped identify eligible participants who met the inclusion criteria (aged 60 years and above, residing in Riyadh, and capable of providing informed consent). Interested participants attended an informational session where they were provided with a study information sheet explaining the research objectives, procedures, and potential risks and benefits. A preliminary screening checklist was then used to confirm eligibility. Participants who met the criteria were asked to sign an informed consent form, after which they were formally enrolled in the study.

Following recruitment, participants completed a structured questionnaire through face-to-face interviews conducted by trained researchers. The Demographic and Health Information Questionnaire was administered first to collect sociodemographic details, medical history, and medication use. Each interview lasted approximately 15–20 min, with rest breaks available upon request. The questionnaire was conducted in Arabic, with simplified explanations provided for any medical terms that participants found difficult to understand. The responses were recorded on digital tablets or paper forms, ensuring accuracy and efficiency in data entry.

To assess physical activity levels, participants completed the International Physical Activity Questionnaire (IPAQ-Elderly Adaptation). Since many older adults have varying literacy levels, the questionnaire was read aloud, and participants verbally provided their responses. The researcher clarified activity types by providing examples of culturally relevant exercises, such as walking for prayer, housework, and traditional physical activities. Each IPAQ interview lasted 10–15 min, and responses were categorized into low, moderate, or high physical activity levels based on the Metabolic Equivalent of Task (MET) scoring system.

After completing the questionnaire, participants underwent frailty and functional capacity assessments using standardized performance-based tests. These assessments were conducted in a quiet and safe environment within the healthcare or community center. The Fried Frailty Phenotype Criteria were used to categorize participants as non-frail, pre-frail, or frail. Frailty assessment included five criteria: unintentional weight loss, exhaustion, grip strength, walking speed, and physical activity level. Weight loss and exhaustion were determined through interview questions, while grip strength was objectively measured using a hand-held dynamometer. Walking speed was assessed using a 4 m walk test, where participants were timed while walking at their usual pace. The total frailty assessment took approximately 15 min per participant.

Functional mobility was further assessed using the Timed Up and Go (TUG) Test. Participants were asked to stand up from a chair, walk 3 m, turn around, and return while being timed. The researcher ensured that participants wore appropriate footwear and that the walking path was clear to prevent falls. The TUG test took approximately 5 min per participant and helped assess balance, coordination, and fall risk. Another functional test, the Chair Stand Test (Five-Times-Sit-to-Stand Test), was conducted to evaluate lower body strength. Participants were instructed to stand up from a chair five times without using their hands, and the total time taken was recorded. This test provided insight into muscle endurance and functional independence.

To measure psychological resilience, participants completed the Connor–Davidson Resilience Scale (CD-RISC-10). This 10-item questionnaire assessed an individual’s ability to cope with stress, maintain optimism, and adapt to challenges. The tool was administered in Arabic, and participants rated each item on a 5-point Likert scale, with total scores ranging from 0 to 40. Higher scores indicated greater psychological resilience. The assessment took approximately 10 min to complete and was facilitated through a structured interview format.

Cognitive function was evaluated using the Mini-Mental State Examination (MMSE), a widely used screening tool for detecting cognitive impairment in older adults. The MMSE included orientation, attention, recall, language, and comprehension tasks. Participants were asked to recall objects, spell words backward, and follow multi-step commands. The test was conducted in Arabic, and cultural modifications were made where necessary. Scoring ranged from 0 to 30, with scores below 24 indicating cognitive impairment. The MMSE assessment took approximately 15 min per participant.

The final phase of data collection involved data entry, verification, and quality control. After all assessments were completed, the research team cross-checked responses and performance test results for completeness and accuracy. Data were entered into a secured database, with unique identification numbers assigned to maintain participant confidentiality. Any missing or unclear responses were clarified by re-contacting the participant or consulting the field notes taken by the research team.

### 2.7. Data Analysis

Data were analyzed with SPSS version 26. Descriptive statistics (means ± standard deviation for continuous variables; frequencies and percentages for categorical variables) were first generated to characterize the sample’s sociodemographic profile, physical-activity categories, resilience scores and frailty status. Group differences in continuous outcomes (e.g., MET-minutes, resilience, grip strength) across gender and age strata were examined with independent samples *t*-tests or one-way analysis of variance (ANOVA) followed by Bonferroni post hoc comparisons, after verifying normality with the Shapiro–Wilk test and homogeneity of variance with Levene’s test. Associations among principal study variables—physical activity, total resilience score, age and a frailty index derived from the number of Fried criteria met—were quantified with Pearson correlation coefficients. To identify independent predictors of frailty, a multivariable logistic-regression model was constructed in which frailty status (frail/pre-frail = 1 vs. non-frail = 0) served as the dependent variable, and physical-activity category, total resilience score, age, gender, body-mass index, comorbidity count, self-rated health, hand-grip strength and MMSE score were simultaneously entered as covariates (full-entry method). Confounders were selected a priori on theoretical and empirical grounds. Model adequacy was assessed with the Hosmer–Lemeshow goodness-of-fit test, and explanatory power with Nagelkerke *R*^2^. Odds ratios (ORs) with 95% confidence intervals (CIs) were reported. Less than four per cent of data were missing; these values were imputed using multiple imputation under a missing-at-random assumption. All tests were two-tailed, and statistical significance was established at *p* < 0.05.

### 2.8. Ethical Consideration

Ethical approval was obtained from the Institutional Review Board (IRB) of the King Saud University in Riyadh (IRB: 25-041) February 2025, ensuring that all study procedures complied with national and international ethical standards for research involving human subjects. Participants were informed about the study’s purpose, the voluntary nature of participation, the confidentiality of their data, and their right to withdraw at any point without repercussions. All data were anonymized by assigning a unique identification code to each participant, and only authorized research team members had access to the password-protected database. The findings of the study will be disseminated to healthcare providers, policymakers, and the academic community, potentially guiding interventions aimed at promoting healthy aging and reducing frailty in older Saudi adults.

## 3. Results

Table 1 presents the sociodemographic and health-related characteristics of the study participants (N = 240). The age distribution indicates that the largest proportion (40%) of participants were in the 70–79 age group, followed by those aged 60–69 years (34%) and those aged 80 years and above (26%). Gender distribution was nearly balanced, with a slight predominance of females (51.3%). Educational attainment varied, with 38.3% of participants having no formal education, highlighting the potential influence of health literacy on aging outcomes. The majority of participants (73.3%) lived with family, reflecting prevailing social structures in Saudi Arabia, while a smaller proportion lived alone (17.5%) or resided in long-term care facilities (9.2%). Regarding body mass index (BMI), two-thirds of participants were either overweight (37.5%) or obese (37.5%), which is consistent with regional epidemiological trends among older adults. In terms of comorbidities, 41.7% of participants reported two to three chronic conditions, while 25% had four or more, underscoring the high burden of multimorbidity in this population. Cognitive status, assessed via the Mini-Mental State Examination (MMSE), revealed that 75% of participants scored within the normal cognitive range, while 25% exhibited mild cognitive impairment. This distribution supports the appropriateness of the inclusion criteria and highlights the need to consider cognitive function in designing interventions for physical activity and frailty prevention.

Table 2 presents the distribution of physical activity levels across gender and age groups. Among participants, 39.6% were categorized as having moderate physical activity, while 39.6% were low and only 20.4% high. Males were more likely to fall in the high-activity category (22%) than females (18.6%). Age-wise, participants aged 60–69 were the most represented in the high-activity group, while low activity was most prevalent among those aged ≥80. These results reflect expected trends in activity decline with age and suggest potential targets for age- and gender-specific physical activity promotion strategies.

In Table 3, “Low Physical Activity” appears both as a criterion under the Fried Frailty Phenotype (FFP) and as a broader category assessed via the International Physical Activity Questionnaire (IPAQ-Elderly). While IPAQ categorizes overall weekly physical activity based on self-reported MET-minutes, the FFP defines low physical activity specifically based on kcal/week thresholds (<383 kcal/week for men, <270 kcal/week for women). Therefore, a participant may be classified as “moderately active” by IPAQ (≥600 MET-min/week) but still meet the FFP frailty criterion for low energy expenditure, reflecting differences in operational definitions and thresholds between tools.

Table 4 displays the subdomain scores of psychological resilience across gender and age categories. Male participants consistently showed slightly higher resilience scores across all domains. Younger age groups (particularly those aged 60–69) demonstrated higher scores in adaptability, optimism, and emotional regulation compared to the ≥80 group, highlighting the decline in psychological flexibility with age. The lowest scoring subdomain overall was optimism, which may warrant targeted intervention strategies focused on improving outlook and self-belief in older populations.

Table 5 illustrates the bivariate correlations among key study variables. As expected, physical activity was positively correlated with resilience (r = 0.61, *p* < 0.001) and negatively correlated with the frailty index (r = −0.59, *p* < 0.001), indicating that more active individuals tended to report higher psychological resilience and fewer frailty indicators. Resilience was also inversely related to frailty (r = −0.51, *p* < 0.001), while age showed a modest positive correlation with frailty (r = 0.41, *p* < 0.05). These associations reinforce the interdependence of physical, psychological, and functional domains in older adults.

Table 6 summarizes the multivariate logistic regression results predicting frailty status. Statistically significant predictors included age, number of comorbidities, poor self-rated health, low physical activity, lower resilience scores, and weaker grip strength. Notably, each unit increase in the resilience score (CD-RISC-10) was associated with a 9% reduction in the odds of being frail (*p* = 0.004). Physical inactivity showed the strongest association, with more than twice the odds of frailty among those in the low activity group (OR = 2.42, *p* = 0.001). Cognitive status (MMSE) approached but did not reach significance. These findings underscore the multifactorial nature of frailty and highlight modifiable factors like physical activity and psychological resilience.

**Figure 1:**
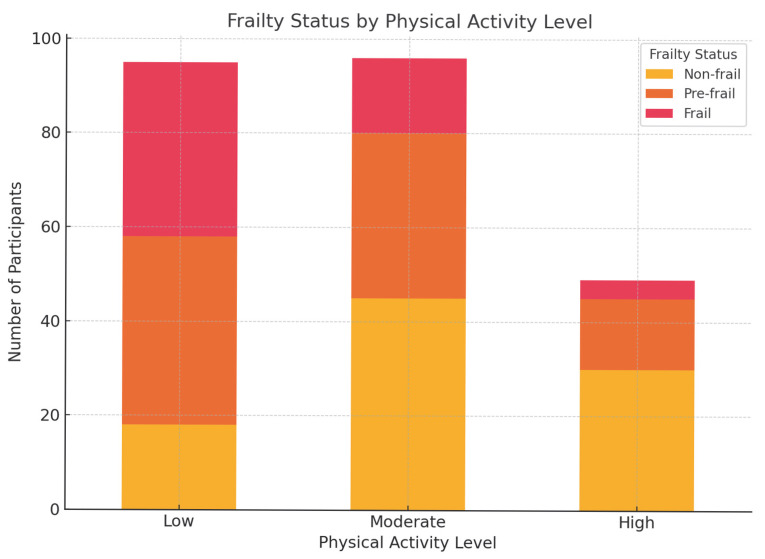
Frailty Status by Physical Activity Level.

Figure 1 presents a visual distribution of frailty status across different levels of physical activity (low, moderate, and high). A clear trend is observed: frailty prevalence decreases as physical activity increases. Among participants classified as having low physical activity, a substantial proportion (approximately 39%) were identified as frail, while only 19% were non-frail. In contrast, the high physical activity group exhibited a predominantly non-frail profile (over 60%), with very few frail individuals (less than 10%). The moderate activity group showed a more balanced distribution, with a notable concentration in the pre-frail category. This figure reinforces the inverse relationship between physical activity and frailty and visually supports the logistic regression finding that low physical activity is a significant predictor of frailty. The graphical representation enhances interpretation and underscores the potential value of promoting physical activity in frailty prevention efforts among older adults.

## 4. Discussion

This study investigated the associations between physical activity, psychological resilience, and frailty among older adults in Riyadh, Saudi Arabia. The findings suggest that higher levels of physical activity and greater resilience are significantly associated with lower frailty risk, whereas advancing age, greater comorbidity burden, poor self-rated health, and reduced grip strength are associated with increased likelihood of frailty. It is important to emphasize that the cross-sectional nature of this study precludes causal inferences. The observed relationships should be interpreted as associations only, and further longitudinal or interventional research is needed to establish causality.

The inverse association between physical activity and frailty aligns with existing evidence underscoring the role of physical activity in preserving muscle strength, balance, and overall functional independence among older adults [6,35]. Notably, the results indicate that participants categorized as having low physical activity levels were more than twice as likely to be frail compared to those in higher activity groups. This finding reinforces the importance of physical activity as a modifiable determinant of frailty. However, discrepancies between the IPAQ-E classifications and the Fried Frailty Phenotype’s criterion for low physical activity were also noted [36]. These inconsistencies may stem from differences in measurement thresholds—IPAQ quantifies total weekly activity in MET-minutes, while the Fried criteria focus specifically on caloric expenditure. This distinction highlights the complexity of assessing physical activity in aging populations and suggests the value of multimodal measurement approaches in future research [37].

Psychological resilience emerged as another strong protective factor against frailty. Participants with higher resilience scores were significantly less likely to be classified as frail, independent of physical health indicators [38,39]. This finding is consistent with the growing literature linking resilience to successful aging and adaptive functioning in later life [40]. A key contribution of the present study lies in the analysis of resilience subdomains. While overall resilience scores were high, *optimism* consistently scored lower than *adaptability* and *emotional regulation* [41,42]. This suggests that while many older adults may feel capable of adapting to life challenges, fewer maintain a strong positive outlook on the future. In the cultural context of Saudi Arabia, where aging is often associated with dependency and reduced social contribution, fostering optimism may require targeted interventions [43]. Structured psychosocial programs—such as cognitive behavioral therapy [44], mindfulness training, and social participation initiatives—have demonstrated efficacy in enhancing optimism and promoting healthy aging [45].

Interestingly, no significant gender differences were found in frailty status or resilience scores. This contrasts with some international studies reporting higher frailty prevalence among women and greater resilience among men [46]. In the Saudi context, however, extended family living arrangements, cultural expectations of elder care, and communal religious practices may buffer gender disparities in psychosocial well-being [47]. Both men and women commonly receive support from family members, including assistance with daily activities, emotional care, and religious engagement, which may enhance perceived support and promote resilience [48]. Moreover, national efforts under Saudi Vision 2030 to promote active aging and inclusive health initiatives may be contributing to the narrowing of gender gaps in physical activity and health behaviors among older adults [49].

Cultural and religious influences play a pivotal role in shaping how older adults in Saudi Arabia experience aging, resilience, and frailty [50]. The concept of *birr al-walidayn* (filial piety), deeply rooted in Islamic and Arab traditions, encourages children to care for aging parents as a moral and religious duty, which often translates into strong family involvement in daily life [51]. This support can serve as a protective factor against social isolation and frailty. Furthermore, religious activities such as mosque attendance, group prayers, and spiritual practices provide older adults with routine, community connection, and a sense of purpose—factors known to enhance resilience and psychological well-being [52]. Interventions designed to reduce frailty and promote resilience in Saudi Arabia should, therefore, consider these cultural values and family-based structures to maximize engagement and effectiveness [53,54].

Despite its strengths, including the use of validated instruments, cultural adaptation of tools, and stratified recruitment from multiple settings, this study has several limitations. First, reliance on self-reported data for physical activity, comorbidities, and resilience introduces the potential for recall and social desirability bias. Although standardized instruments like the IPAQ and CD-RISC were used, some over- or under-reporting may have occurred. Second, the MMSE, while a widely used screening tool for cognitive function, may not detect subtle cognitive impairments, especially among individuals with limited formal education [55]. Third, comorbidities were self-reported and not confirmed via medical records, which may have led to underestimation or misclassification. Fourth, the use of convenience sampling and non-random site selection limits the generalizability of findings to the broader older adult population in Saudi Arabia. Nevertheless, the inclusion of participants from diverse care settings—community centers, primary healthcare facilities, and long-term care homes—adds to the ecological validity of the findings.

In summary, this study highlights the interconnected roles of physical activity and psychological resilience in protecting against frailty among older adults. The findings reinforce the importance of addressing both physical and psychological health domains in interventions aimed at promoting healthy aging. Cultural and religious factors, particularly family support and spiritual engagement, may act as important mediators and should be considered in the development of context-specific health promotion strategies. Future research should utilize longitudinal designs to confirm these associations over time and evaluate the impact of integrated, culturally tailored interventions that enhance both physical activity and psychological resilience [56].

### Limitations of the Study

Despite providing valuable insights into the relationship between physical activity, resilience, and frailty in older adults, this study has several notable limitations. First, the cross-sectional design restricts the ability to make causal inferences. Although physical activity and resilience are associated with reduced frailty, the temporal sequence remains unclear—longitudinal or interventional studies are needed to establish causality. Second, convenience sampling was employed, which may limit the generalizability of the findings to all older adults in Riyadh or other regions of Saudi Arabia. Third, the study relied on self-reported questionnaires for certain measures, raising the possibility of recall bias and social desirability bias, where participants may have over- or underreported their physical activity levels. Fourth, cognitive function and comorbid conditions were assessed through brief screening tools and self-report, which may not capture the full complexity of medical or cognitive status. Finally, cultural factors unique to Saudi Arabia (e.g., family support structures, gender norms) may influence outcomes differently compared to Western or other Middle Eastern contexts, potentially limiting global applicability.

## 5. Conclusions

In conclusion, this study underscores the significance of physical activity and psychological resilience as protective factors against frailty in older adults residing in Riyadh. Higher activity levels and robust resilience emerged as independent predictors of reduced frailty, suggesting a multidimensional approach to healthy aging that includes regular exercise and emotional well-being interventions. Concurrently, advancing age, poor self-rated health, and multiple comorbidities surfaced as notable risk factors for frailty, emphasizing the importance of holistic healthcare strategies tailored to the specific needs of older individuals. By integrating comprehensive medical assessments, structured physical activity programs, and interventions aimed at strengthening resilience, healthcare providers can more effectively mitigate frailty and enhance quality of life for the elderly population. Future research, particularly longitudinal and interventional studies, is warranted to clarify the temporal relationships and to develop evidence-based guidelines that can be applied across diverse cultural and healthcare settings.

## Figures and Tables

**Table 1:** Demographic Characteristics of Participants (N = 240).

Variable	Category	Frequency (n)
Age Group	60–69	82
	70–79	96
	≥80	62
Gender	Male	117
	Female	123
Education	No formal education	92
	Primary	48
	Secondary	62
	Higher	38
Living Arrangement	Alone	42
	With family	176
	LTCF	22
Body Mass Index (BMI)	<25 (Normal)	60
	25–29.9 (Overweight)	90
	≥30 (Obese)	90
Number of Comorbidities	0–1	80
	2–3	100
	≥4	60
Cognitive Status (MMSE)	Normal (24–30)	180
	Mild impairment (18–23)	60

**Table 2:** Distribution of Physical Activity Levels and Mean Weekly MET-minutes, by Gender (N = 240).

Physical Activity Level	Total (n)	Male (n)	Female (n)	60–69 (n)	70–79 (n)	≥80 (n)	Mean MET-min/Week ± SD
Low	95	48	47	20	37	38	684.2 ± 146.9
Moderate	96	51	45	42	38	16	1683.4 ± 271.8
High	49	28	21	20	21	8	2594.3 ± 341.7
Total	240	127	113	82	96	62	1694.8 ± 297.6

Note: IPAQ-Elderly classification used to assess physical activity categories. MET = Metabolic Equivalent of Task.

**Table 3:** Distribution of Fried Frailty Phenotype Criteria and Overall Frailty Classification (N = 240).

Frailty Component	Yes (n)	No (n)	Total (n)
Unintentional Weight Loss	36	204	240
Exhaustion	71	169	240
Weak Grip Strength	58	182	240
Slow Walking Speed	44	196	240
Low Physical Activity *	95	145	240
Overall Frailty Status			
Non-frail (0 criteria)			94
Pre-frail (1–2 criteria)			88
Frail (≥3 criteria)			58

* Low physical activity status here is based on Fried’s threshold, which may differ from the IPAQ categories.

**Table 4:** Descriptive Statistics for Resilience Subdomains and Total Resilience (N = 240).

Resilience Domain	Total Mean ± SD	Male (Mean ± SD)	Female (Mean ± SD)	60–69 (Mean)	70–79 (Mean)	≥80 (Mean)
Adaptability (4 items)	13.7 ± 2.2	13.9 ± 2.1	13.5 ± 2.3	14.1	13.8	13.1
Optimism (3 items)	10.9 ± 1.8	11.1 ± 1.7	10.7 ± 1.9	11.2	10.8	10.5
Emotional Regulation (3 items)	11.6 ± 2.1	11.8 ± 2.2	11.3 ± 2.0	12.0	11.5	11.0
Total CD-RISC-10	36.2 ± 4.1	36.8 ± 3.9	35.6 ± 4.3	37.3	36.1	34.6

Note: CD-RISC-10 subdomains were derived from factor analysis of the 10-item resilience scale: Adaptability: Ability to adjust to change and persist in the face of adversity. Optimism: A positive outlook and confidence in overcoming hardship. Emotional Regulation: Ability to manage emotions under stress.

**Table 5:** Correlation Matrix Among Key Variables.

Variables	Physical Activity	Resilience Score	Age	Frailty Index
Physical Activity	–	0.61 **	−0.27 *	−0.59 **
Resilience Score		–	−0.14	−0.51 **
Age			–	0.41 *
Frailty Index				–

Note: PA = Physical Activity (MET-min/week, IPAQ); Resilience Score = Total score from CD-RISC-10; FI = Frailty Index (sum of Fried criteria met). * *p* < 0.05, ** *p* < 0.001 (2-tailed).

**Table 6:** Multiple Logistic Regression Predicting Frailty Status (N = 240).

Predictor Variable	Odds Ratio (OR)	95% CI	*p*-Value
Age (years)	1.08 *	1.03–1.16	0.012
Gender (female)	1.23	0.75–2.01	0.312
BMI (kg/m^2^)	1.05	0.97–1.13	0.218
Number of Comorbidities	1.36 *	1.10–1.68	0.005
Self-rated Health (poor)	1.88 *	1.13–3.15	0.017
Physical Activity (low)	2.42 *	1.40–4.21	0.001
Resilience Score (CD-RISC-10)	0.91 *	0.86–0.97	0.004
Grip Strength (kg)	0.93 *	0.89–0.98	0.008
MMSE Score	0.95	0.89–1.01	0.084

Significant at * *p* < 0.05. OR = odds ratio; CI = confidence interval; BMI = body mass index; MMSE = Mini-Mental State Examination. Dependent variable: Frailty status (frail/pre-frail = 1, non-frail = 0).

## Data Availability

Data are available within the manuscript.
